# Identification of lncRNA expression profile in the spinal cord of mice following spinal nerve ligation-induced neuropathic pain

**DOI:** 10.1186/s12990-015-0047-9

**Published:** 2015-07-17

**Authors:** Bao-Chun Jiang, Wen-Xing Sun, Li-Na He, De-Li Cao, Zhi-Jun Zhang, Yong-Jing Gao

**Affiliations:** Pain Research Laboratory, Institute of Nautical Medicine, Jiangsu Key Laboratory of Inflammation and Molecular Drug Target, Nantong University, 9 Seyuan Road, Nantong, 226019 Jiangsu China; Co-innovation Center of Neuroregeneration, Nantong University, Nantong, 226001 Jiangsu China; Department of Nutrition and Food Hygiene, School of Public Health, Nantong University, Nantong, 226001 Jiangsu China

**Keywords:** LncRNA, Spinal cord, Spinal nerve ligation, Neuropathic pain

## Abstract

**Background:**

Neuropathic pain that caused by lesion or dysfunction of the nervous system is associated with gene expression changes in the sensory pathway. Long noncoding RNAs (lncRNAs) have been reported to be able to regulate gene expression. Identifying lncRNA expression patterns in the spinal cord under normal and neuropathic pain conditions is essential for understanding the genetic mechanisms behind the pathogenesis of neuropathic pain.

**Results:**

Spinal nerve ligation (SNL) induced rapid and persistent pain hypersensitivity, characterized by mechanical allodynia and heat hyperalgesia. Meanwhile, astrocytes and microglia were dramatically activated in the ipsilateral spinal cord dorsal horn at 10 days after SNL. Further lncRNA microarray and mRNA microarray analysis showed that the expression profiles of lncRNA and mRNA between SNL and sham-operated mice were greatly changed at 10 days. The 511 differentially expressed (>2 fold) lncRNAs (366 up-regulated, 145 down-regulated) and 493 mRNAs (363 up-regulated, 122 down-regulated) were finally identified. The expression patterns of several lncRNAs and mRNAs were further confirmed by qPCR. Functional analysis of differentially expressed (DE) mRNAs showed that the most significant enriched biological processes of up-regulated genes in SNL include immune response, defense response, and inflammation response, which are important pathogenic mechanisms underlying neuropathic pain. 35 DE lncRNAs have neighboring or overlapping DE mRNAs in genome, which is related to Toll-like receptor signaling, cytokine–cytokine receptor interaction, and peroxisome proliferator-activated receptor signaling pathway.

**Conclusion:**

Our findings uncovered the expression pattern of lncRNAs and mRNAs in the mice spinal cord under neuropathic pain condition. These lncRNAs and mRNAs may represent new therapeutic targets for the treatment of neuropathic pain.

## Background

Neuropathic pain is one of the most common chronic pain in humans and characterized by an increase in the responsiveness of nociceptive neurons in the peripheral and central nervous system (CNS) [1]. Peripheral and central sensitization represents the altered functional status of nociceptive neurons and results from changes of a vast amount of functional protein and signaling pathways in the neuron and glial cell [2, 3]. Recent pharmaceutical research and discovery activities focus on well-characterized molecular targets, such as ion channels, G-protein-coupled receptors, and kinases in neurons and glial cells localized along the nociceptive pathways, which are regarded as direct contributors to the sensitization of pain signaling systems [4, 5]. However, the transcriptional or translational regulatory mechanisms underlying the expression and functional changes of these molecules are poorly defined.

RNAs that do not code for a protein (noncoding RNAs, ncRNAs) consist of two major classes: the small ncRNAs, which include microRNAs (miRNAs) and other noncoding transcripts of less than 200 nucleotides, and long noncoding RNAs (lncRNAs), which are a novel class of non-protein coding transcripts longer than 200 nucleotides [6]. LncRNAs were initially considered as transcriptional by-products, but recent data suggest that lncRNAs can regulate gene expression via interfering with transcription, post-transcriptional processing, chromatin remodeling, miRNA sequestration, and generating small ncRNAs [7, 8]. Also, lncRNAs are involved in various aspects of cell biology and disease etiology, such as development [9], immune [10], cardiovascular disease [11], oncogenesis [12], and neurological disease [13]. LncRNAs are highly expressed in the CNS, and their expression profiles are associated with specific neuroanatomical regions, cell types, or subcellular compartments suggesting their potential functional roles in the nervous system [14–16]. It was reported that sciatic nerve resection induced differential expression of lncRNAs in dorsal root ganglia (DRG) [17]. Moreover, Zhao et al. have recently identified a functional lncRNA Kcna2, which contributed to neuropathic pain by silencing Kcna2 in DRG neurons [18]. These findings indicate the involvement of lncRNAs in neuropathic pain.

The spinal cord is responsible for receiving input from nociceptors and projecting to the brain, and plays an important role in the integration and modulation of pain-related signals. To clarify the molecular mechanisms underlying neuropathic pain and explore novel approaches for analgesic strategies, herein, we investigated the genome-wide expression of lncRNAs in the spinal cord following L5 spinal nerve ligation (SNL)-induced neuropathic pain. We found a large number of differentially expressed (DE) lncRNAs and mRNAs in the spinal cord after SNL. Among them, 39 correlated lncRNA-mRNA pairs, consisting of DE lncRNAs and mRNAs with adjacent or overlapping position relationship, were screened out. Our findings will provide new insights into the roles of lncRNAs in the regulation of neuropathic pain-associated genes.

## Results

### Model identification of neuropathic pain

**Figure 1:**
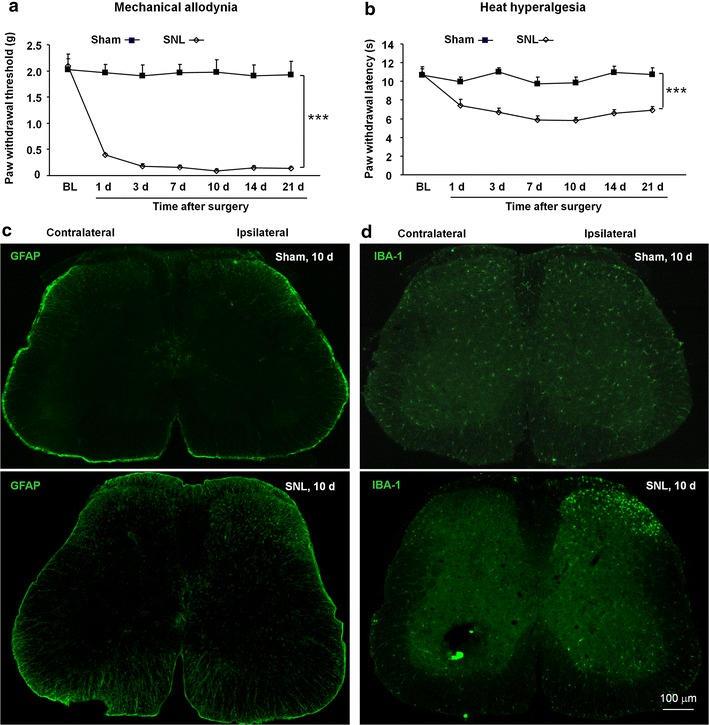
SNL induces persistent neuropathic pain and glial activation in the spinal cord. SNL-induced rapid and persistent mechanical allodynia (**a**) and heat hyperalgesia (**b**). Data are expressed as mean ± SEM (n = 5 for each group). ***P < 0.001, two-way repeated measures ANOVA. **c**, **d** Representative images of GFAP and IBA-1 immunofluorescence in the L5 spinal cord from sham and SNL mice. GFAP and IBA-1 immunoreactive were very low in sham-treated mice, but significantly increased in the ipsilateral superficial dorsal horn at 10 days after SNL.

The SNL model has been widely used in the investigation of the mechanisms underlying neuropathic pain [19]. Here we also found that SNL induced rapid (1 d) and persistent (>21 d) mechanical allodynia (Figure 1a) and heat hyperalgesia (Figure 1b) in mice. We then harvested the spinal cord at 10 days (maintenance phase) after SNL and checked the expression of astrocytic marker GFAP and microglial marker IBA-1, which are known to be upregulated in the spinal cord under neuropathic pain condition [20, 21]. As shown in Figure 1c, d, GFAP expression and IBA-1 expression were both increased in the ipsilateral dorsal horn in SNL animals but not in sham-treated animals, indicating that glial activation was induced in the spinal cord by SNL.

### Overview of lncRNAs and mRNA expression profiles after SNL

**Figure 2:**
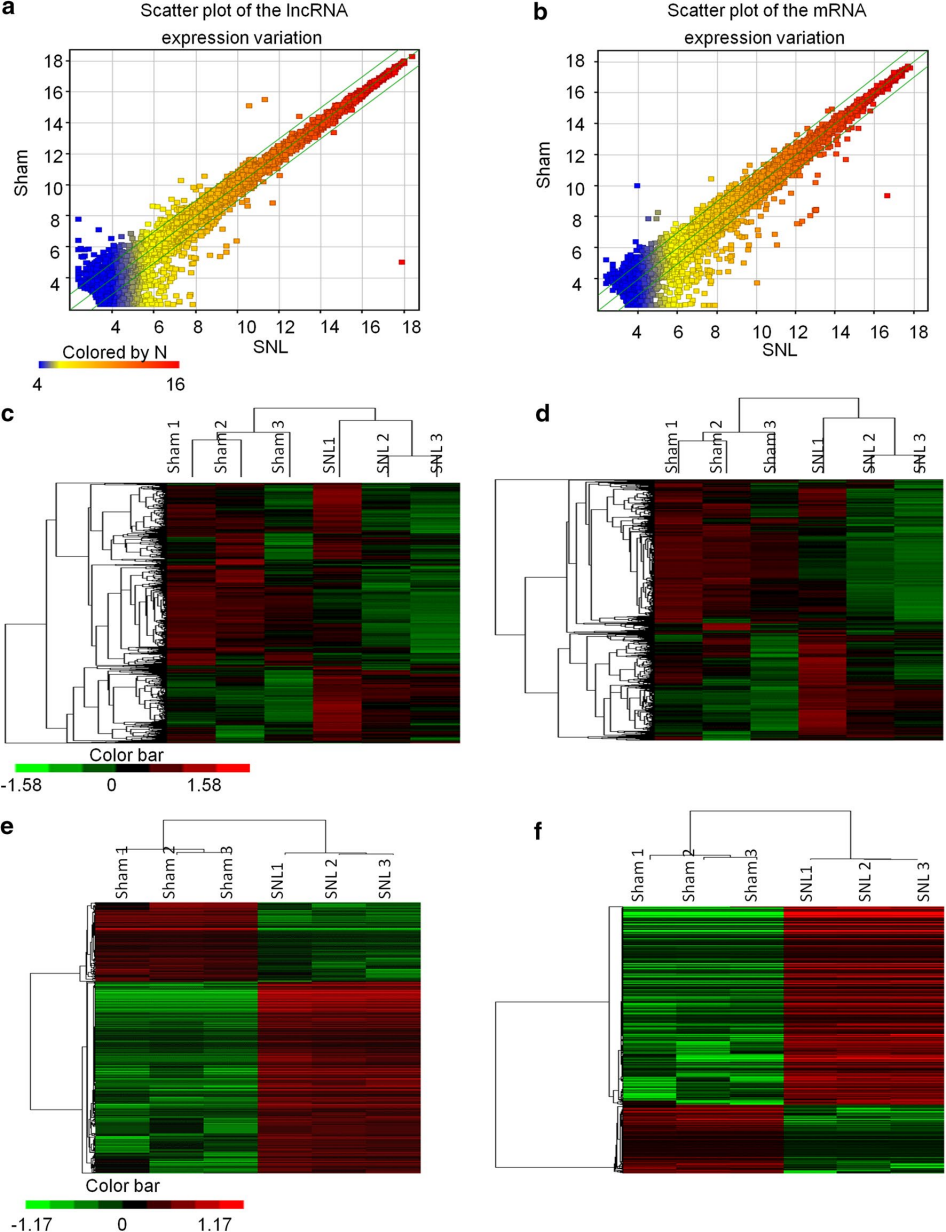
SNL results in the expression profiling changes of lncRNA and mRNA. Scatter plot comparing global lncRNA (**a**) or mRNA (**b**) gene expression profiles in the spinal cord between the SNL and sham mice. *Green lines* indicate twofold differences in either direction in lncRNA and mRNA expression. Heat map showing hierarchical clustering of overall lncRNAs (**c**) or mRNA (**d**) expression pattern of reliably measured probe sets. Heat map showing hierarchical clustering of LncRNAs (**e**) or mRNA (**f**), whose expression changes were more than twofold. In clustering analysis, up- and down-regulated genes are *colored* in *red* and *green*, respectively.

We then detected the expression profiles of lncRNAs and mRNAs in the L5 spinal cord at 10 days after SNL by microarray. First, we obtained a graphically overview of the expression signatures of lncRNAs and mRNAs by using scatter plot and hierarchical clustering analyses. The scatter plots showed that a large number of lncRNAs and mRNAs were differentially expressed between SNL and sham-operated mice (Figure 2a, b). Hierarchical cluster analysis of all lncRNAs or mRNA showed that the 3 sham or 3 SNL samples were clustered together respectively, and signal intensity was consistent in sham or SNL group (Figure 2c, d). The heatmap of DE lncRNAs or mRNAs whose expression were up-regulated or down-regulated by twofold were magnified (Figure 2e, f), indicating the high level of concordance in either SNL or sham samples. These data suggest that neuropathic pain is associated with the changes of lncRNAs and mRNAs in the spinal cord.

### Differentially expressed lncRNAs and mRNAs

We further analyzed differentially expressed (DE) lncRNAs using significance analysis of microarrays method, following the criteria q-value <0.05, and fold change >2. The results showed that 511 lncRNAs, containing 366 up-regulated and 145 down-regulated, were significantly changed in SNL group, comparing with the sham group. The most up-regulated lncRNAs were: *uc009egw.1*, *Speer7*-*ps1*, *MM9LINCRNAEXON12113*+, *ENSMUST00000118074*, and *uc009nzx.1*, of which *uc009egw.1* showed the largest up-regulation (Log_2_ fold change = 7,332.4243). The most down-regulated lncRNAs were: *AK045739*, *AK020832*, *AK047380*, *ENSMUST00000171761* and *uc008dwx.1*, of which *AK045739* showed the largest down-regulation (Log_2_ fold change = −45.320816). Detailed information including the top 20 up-regulated and 20 down-regulated lncRNAs was listed in Table 1.

**Table 1:** The detail information of the top 20 up-regulated and 20 down-regulated lncRNAs

Up-regulated lncRNAs	Log2 fold change (SNL/sham)	P-value	Down-regulated lncRNAs	Log2 fold change (SNL/sham)	P-value
*uc009egw.1*	7,332.4243	7.07E−08	*AK045739*	−45.320816	4.4E−09
*Speer7-ps1*	44.854053	3.44E−10	*AK020832*	−17.557217	4.08E−09
*MM9LINCRNAEXON12113+*	28.60862	0.000138	*AK047380*	−13.752911	0.0000278
*ENSMUST00000118074*	27.38603	0.0000454	*ENSMUST00000171761*	−11.646089	7.18E−09
*uc009nzx.1*	26.05991	8.21E−06	*uc008dwx.1*	−10.924568	0.00000859
*ENSMUST00000165428*	25.460197	0.0000126	*AK134918*	−9.165875	0.02724757
*CJ300890*	23.606705	4.64E−06	*ENSMUST00000160545*	−8.490716	0.0000666
*MM9LINCRNAEXON11661+*	20.514269	7.95E−08	*AK013492*	−8.088422	0.000000162
*CJ059670*	19.443495	1.31E−07	*MM9LINCRNAEXON10414*−	−7.6248446	0.000158
*NR_003548*	18.795507	7.68E−08	*CA874578*	−6.9701576	0.017664054
*AK086225*	17.855263	0.0000016	*AK045554*	−6.809595	0.000000323
*ENSMUST00000122927*	15.532128	0.00000174	*uc007cua.1*	−6.378551	0.0000552
*ENSMUST00000150343*	14.018134	0.00000125	*NR_030776*	−5.654923	0.00000175
*ENSMUST00000120145*	13.556047	0.000000275	*MM9LINCRNAEXON12090+*	−4.920551	0.000137
*MM9LINCRNAEXON10692+*	13.423789	0.00000243	*MM9LINCRNAEXON10317+*	−4.7705894	0.00000728
*ENSMUST00000121611*	13.380642	0.00000161	*uc007kom.1*	−4.603345	5.78E−09
*humanlincRNA1606+*	13.149129	0.0000756	*ENSMUST00000134042*	−4.242255	0.0000337
*AK085402*	12.824574	0.000378	*ENSMUST00000040306*	−4.188862	0.00000142
*ENSMUST00000121062*	12.615327	0.000000185	*AK157618*	−4.0846663	0.000106
*AK044525*	12.42756	0.00000111	*MM9LINCRNAEXON12066*−	−4.066157	0.00000917

In the DE mRNAs, there are 493 genes whose mRNA change was more than twofold, and the number of up-regulated (363) mRNAs was larger than down-regulated (122) mRNAs in SNL. These DE mRNAs contain many known genes involving in pain processing, including *Cacna1g* (calcium channel, voltage-dependent, T type, alpha 1G subunit, 16.0978 fold increase) [22], *Trpv1* (transient receptor potential cation channel, subfamily V, member 1, 9.31-fold increase) [23], *Ccl5* (chemokine (C-C motif) ligand 5, 3.93-fold increase) [24], *Cx3cr1* (chemokine (C-X3-C) receptor 1, 2.51-fold increase) [25], and *Irf5* (interferon regulatory factor 5) [26]. Besides, a lot of other genes, whose roles in pain have not been identified, were dramatically changed. Further analysis showed that 39 genes whose expression were changed >tenfold, including 38 up-regulated genes and 1 down-regulated gene, such as *Sprr1a* (small proline-rich protein 1A, 148.7-fold), *Anxa10* (annexin A10, 76.3-fold), and *Kng1* (kininogen 1, 38.4-fold); 66 genes whose expression was changed between 5- and 10-fold, including 64 up-regulated and 2 down-regulated genes. Detailed information about the top 20 up-regulated and 20 down-regulated mRNAs was listed in Table 2.

**Table 2:** The detail information of the top 20 up-regulated and 20 down-regulated mRNAs

Gene symbol		Description	Log_2_ fold change (SNL/sham)	*P*-value
Up-regulated genes				
* Sprr1a*		Small proline-rich protein 1A	148.7115	1.84E−10
* Anxa10*		Annexin A10	76.262054	1.61E−06
* 4933402N22Rik*		RIKEN cDNA 4933402N22 gene	46.512726	1.62E−10
* Vmn2r101*		Vomeronasal 2, receptor 101	44.090027	1.2E−08
* Kng1*		Kininogen 1	38.42939	2.14E−08
* Olfr803*		Olfactory receptor 803	31.403961	7.82E−08
* Gpr151*		G protein-coupled receptor 151	27.673513	5.95E−11
* LOC100048884*		Novel member of the major urinary protein (Mup) gene family	24.719683	9.12E−09
* Mup11*		Major urinary protein 11	24.027332	8.26E−10
* Mup7*		Major urinary protein 7	23.950233	2.18E−08
* Mup12*		Major urinary protein 12	23.768707	2.79E−10
* Mup13*		Major urinary protein 13	23.234575	9.99E−08
* Mup19*		Major urinary proteins 11 and 8	23.019644	0.000000314
* Mup8*		Major urinary protein 8	22.686306	0.000000241
* Mup17*		Major urinary protein 17	21.82689	8.07E−10
* Atf3*		Activating transcription factor 3	19.8067	0.00000165
* Rreb1*		Ras responsive element binding protein 1	19.512457	0.0000258
* Olfr648*		Olfactory receptor 648	19.249556	0.00000434
* Clps*		Colipase, pancreatic	18.952599	0.000000801
* Vax2*		Ventral anterior homeobox containing gene 2	17.30259	0.000187
Down-regulated genes				
* Lefty1*		Left right determination factor 1	−10.109003	0.000000123
* Olfr866*		Olfactory receptor 866	−7.406356	0.011693356
* Kcna5*		Potassium voltage-gated channel, shaker-related subfamily, member 5	−5.9395947	0.0000537
* Tnnt2*		Troponin T2, cardiac	−4.8715253	0.000213
* Csprs*		Component of Sp100-rs	−4.639864	0.000183
* Gm5458*		Predicted gene 5458	−3.9395294	0.000162
* Ypel4*		Yippee-like 4 (Drosophila)	−3.8847303	0.0000976
* Sell*		Selectin, lymphocyte	−3.7625916	0.000967
* Mnx1*		Motor neuron and pancreas homeobox 1	−3.702038	0.003540842
* Fnip1*		Folliculin interacting protein 1	−3.4727607	0.000226
* Epm2a*		Epilepsy, progressive myoclonic epilepsy, type 2 gene alpha	−3.363634	0.00031
* H2-Ea-ps*		Histocompatibility 2, class II antigen E alpha, pseudogene	−3.2939498	0.000021
* Chodl*		Chondrolectin	−3.2821681	0.00000249
* Wtap*		Wilms’ tumour 1-associating protein	−3.1569881	0.0000001
* Pira4*		Paired-Ig-like receptor A4	−3.1222947	0.03241746
* Eml4*		Echinoderm microtubule associated protein like 4	−3.117333	0.020077666
* Tnnt2*		Troponin T2, cardiac	−3.0204759	0.0001
* Retnlg*		Resistin like gamma	−2.9266624	0.000000051
* Mmp8*		Matrix metallopeptidase 8	−2.9234846	0.000255

### Real-time quantitative PCR (qPCR) validation of lncRNA and mRNA expression

**Figure 3:**
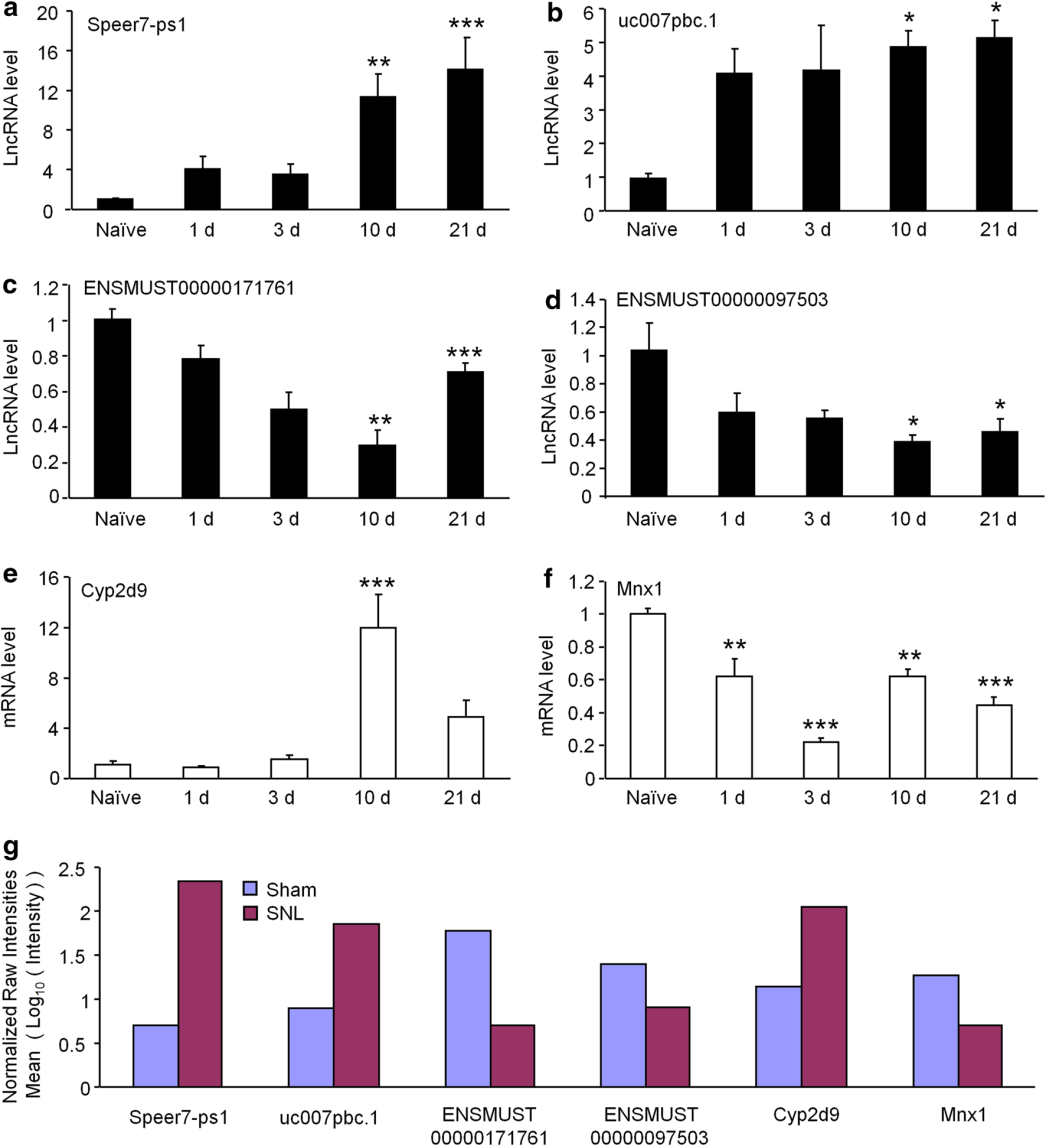
QPCR validations of four deregulated lncRNAs and two deregulated mRNA in the spinal cord from SNL mice. The expressions of lncRNA *Speer7*-*ps1* (**a**), lncRNA *Uc007pbc.1* (**b**), lncRNA *ENSMUST00000171761* (**c**), and lncRNA *ENSMUST00000097503* (**d**) were significantly deregulated at 10 and 21 days after SNL. **e** The expression of *Cyp2d9* mRNA was markedly up-regulated at 10 days after SNL. **f** The expression of *Mnx1* mRNA was significantly down-regulated at 1, 3, 10 and 21 days after SNL. One-way ANOVA followed by Tukey’s multiple comparison test. *P < 0.01, **P < 0.01, ***P < 0.001. **g **Log_ 10_ value of signal intensity detected by microarray.

To validate the reliability of the microarray results and also analyze the temporal changes of lncRNA and mRNA expression after SNL, the up-regulated lncRNAs including *Speer7*-*ps1* and *uc007pbc.1*, the down-regulated lncRNAs, including *ENSMUST00000171761* and *ENSMUST00000097503*, the up-regulated mRNA *Cyp2d9*, and the down-regulated mRNA *Mnx1* were randomly selected and analyzed by qPCR. The spinal cord tissues were collected from naïve animals, and SNL animals at 1, 3, 10, and 21 days. *Speer7*-*ps1* and *uc007pbc.1*, which are intergenic lncRNAs, were both significantly increased at 10 days and peaked at 21 days (Figure 3a, b). *ENSMUST00000171761* and *ENSMUST00000097503* are antisense overlap and bidirectional lncRNA with matching gene *Tagap* (T-cell activation Rho GTPase-activating protein) and *Zfp236* (zinc finger protein 236). They were significantly decreased at 10 days and persisted to 21 days (Figure 3c, d). *Cyp2d9*, a member of cytochrome P450, family 2, subfamily d, was increased more than 12-fold at 10 days (Figure 3e). *Mnx1* is a sequence-specific DNA binding transcription factor. It decreased from 1 to 21 days (Figure 3f). In addition, the fold changes of these lncRNAs and mRNAs detected by qPCR at SNL 10 days were consistent with the results from microarray (Figure 3g), further supporting the reliability of the array data.

### Class distribution of changed LncRNAs

**Figure 4:**
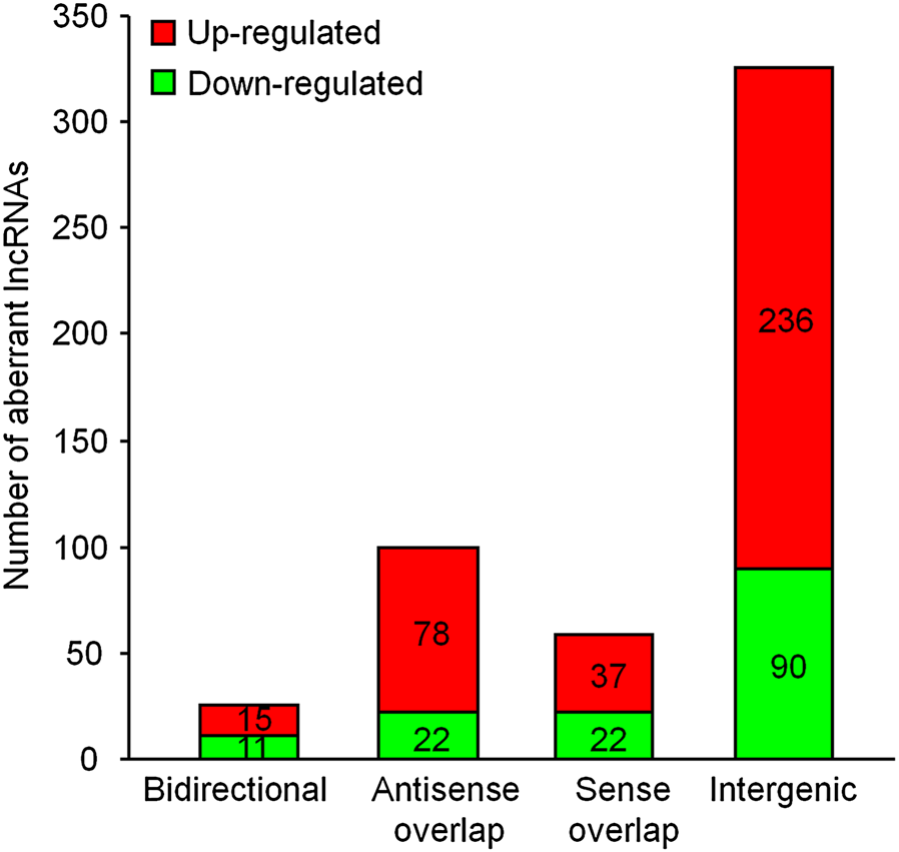
Distribution of various types of DE lncRNAs. Four classes (sense overlap lncRNAs, antisense overlap lncRNAs, bidirectional lncRNAs and intergenic lncRNAs) were analyzed.

lncRNAs were shown to regulate the expression of adjacent or overlapping mRNAs in genome [18, 27, 28]. Thus, the associations of DE lncRNAs with coding genes were analyzed and classified according to the method described by Li et al. [29]. LncRNAs are classified into four groups: intergenic lncRNAs (lncRNAs are located and transcribed from intergenic regions, and do not overlap with known protein coding genes or other types of genes in genome. It is also called lincRNAs), antisense lncRNAs (LncRNA exon is transcribed from the antisense strand and overlaps with a coding transcript exon), sense lncRNAs (LncRNA exon overlaps with a coding transcript exon on the same genomic strand), and bidirectional lncRNAs (LncRNA is oriented head to head with a coding transcript within 1,000 bp). As shown in Figure 4, among the DE lncRNAs, intergenic lncRNAs were the largest category, with 236 up-regulated and 90 down-regulated lncRNAs. The other DE lncRNAs included 100 antisense lncRNAs (78 up-regulated and 22 down-regulated), 59 sense lncRNAs (37 up-regulated and 22 down-regulated), and 26 bidirectional lncRNAs (15 up-regulated and 11 down-regulated).

### Functional prediction of DE mRNAs in SNL

**Figure 5:**
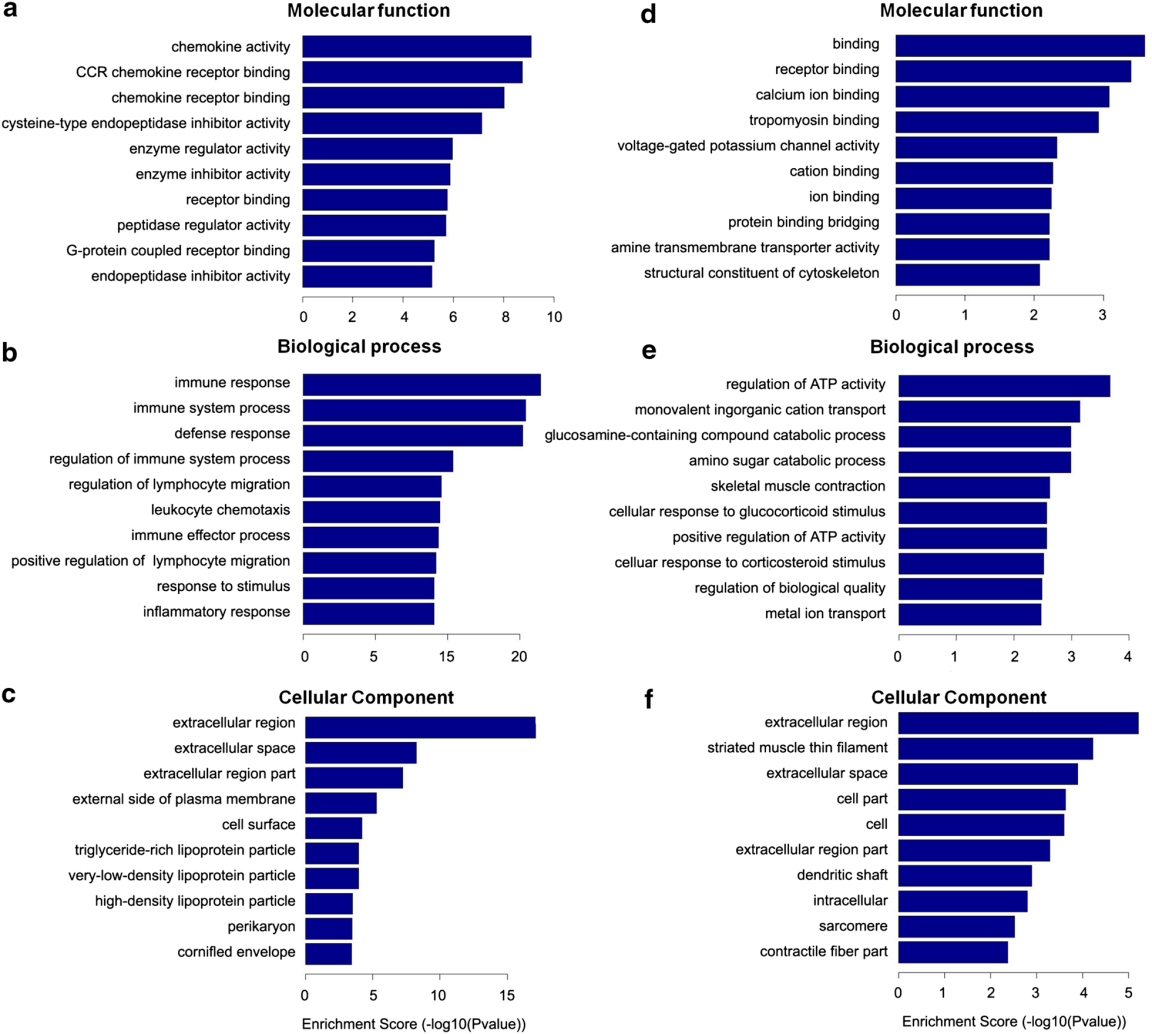
Biological functions of differentially expressed mRNAs with fold changes >2. **a**–**c** The significant molecular function, biological process and cellular component of up-regulated mRNAs. **d**–**f** The significant molecular function, biological process and cellular component of down-regulated mRNAs.

To explore the molecular mechanism in neuropathic pain, we further did GO and pathway analysis of deregulated genes in SNL versus sham. The GO results showed that the most significant enriched molecular function of up-regulated genes in SNL was chemokine activity, CCR chemokine receptor binding, chemokine receptor binding, and cysteine-type endopeptidase inhibitor activity (Figure 5a). The most significant enriched biological processes of up-regulated genes in SNL were immune response, immune system process, defense response, and regulation of immune system process (Figure 5b). The most noteworthy enriched cellular components of up-regulated genes in SNL were extracellular region, extracellular space, extracellular region part, and external side of plasma membrane (Figure 5c). The most significant enriched molecular function of down-regulated genes in SNL were binding, receptor binding, calcium ion binding, and tropomyosin binding (Figure 5d). The most significant enriched biological processes of down-regulated genes in SNL were regulation of ATPase activity, monovalent inorganic cation transport, glucosamine-containing compound catabolic process, and amino sugar catabolic process (Figure 5e). The most significant enriched cellular components of down-regulated genes in SNL were extracellular region, striated muscle thin filament, extracellular space, and cell part (Figure 5f).

**Figure 6:**
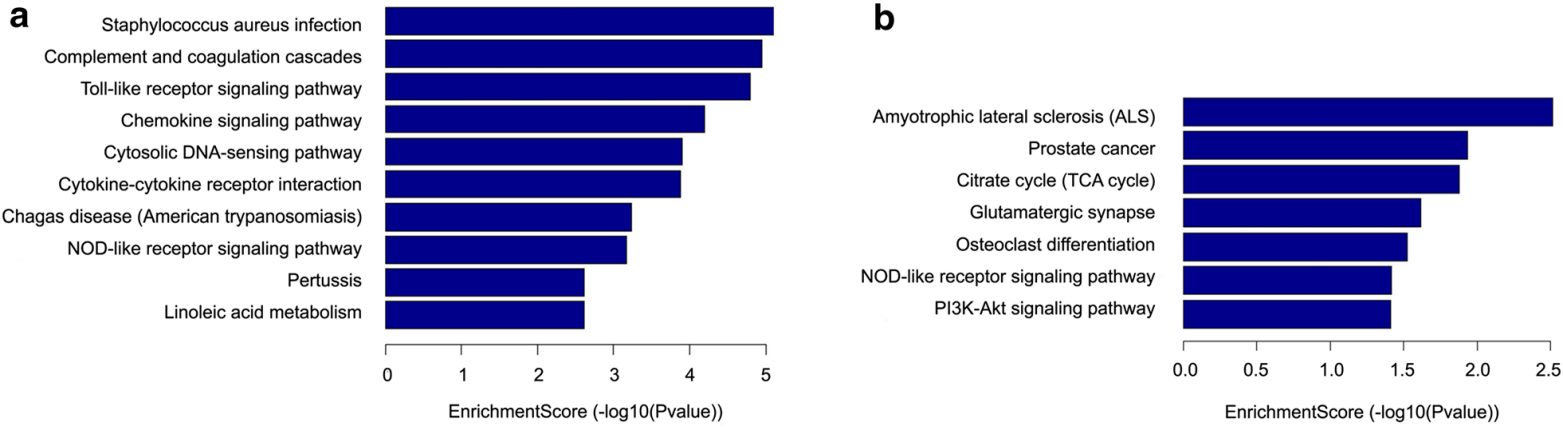
Pathway analysis for 366 up-regulated and 127 down-regulated mRNAs with fold changes >2. **a** The significant pathways for up-regulated genes in SNL group. **b** The significant pathways for down-regulated genes in SNL group.

Similarly, different genes were analyzed in KEGG. The results showed that the up-regulated genes in SNL are involved in complement and coagulation cascades, Toll-like receptor signaling pathway, chemokine signaling pathway, cytosolic DNA-sensing pathway, and cytokine–cytokine receptor interaction, Changas disease, and NOD-like receptor signaling pathway (Figure 6a). Down-regulated genes in SNL are involved in amyotrophic lateral sclerosis (ALS), prostate cancer, citrate cycle, glutamatergic synapse, osteoclast differentiation and NOD-like receptor signaling pathway (Figure 6b).

### Comparison of our DE mRNAs with previously published microarrays

**Figure 7:**
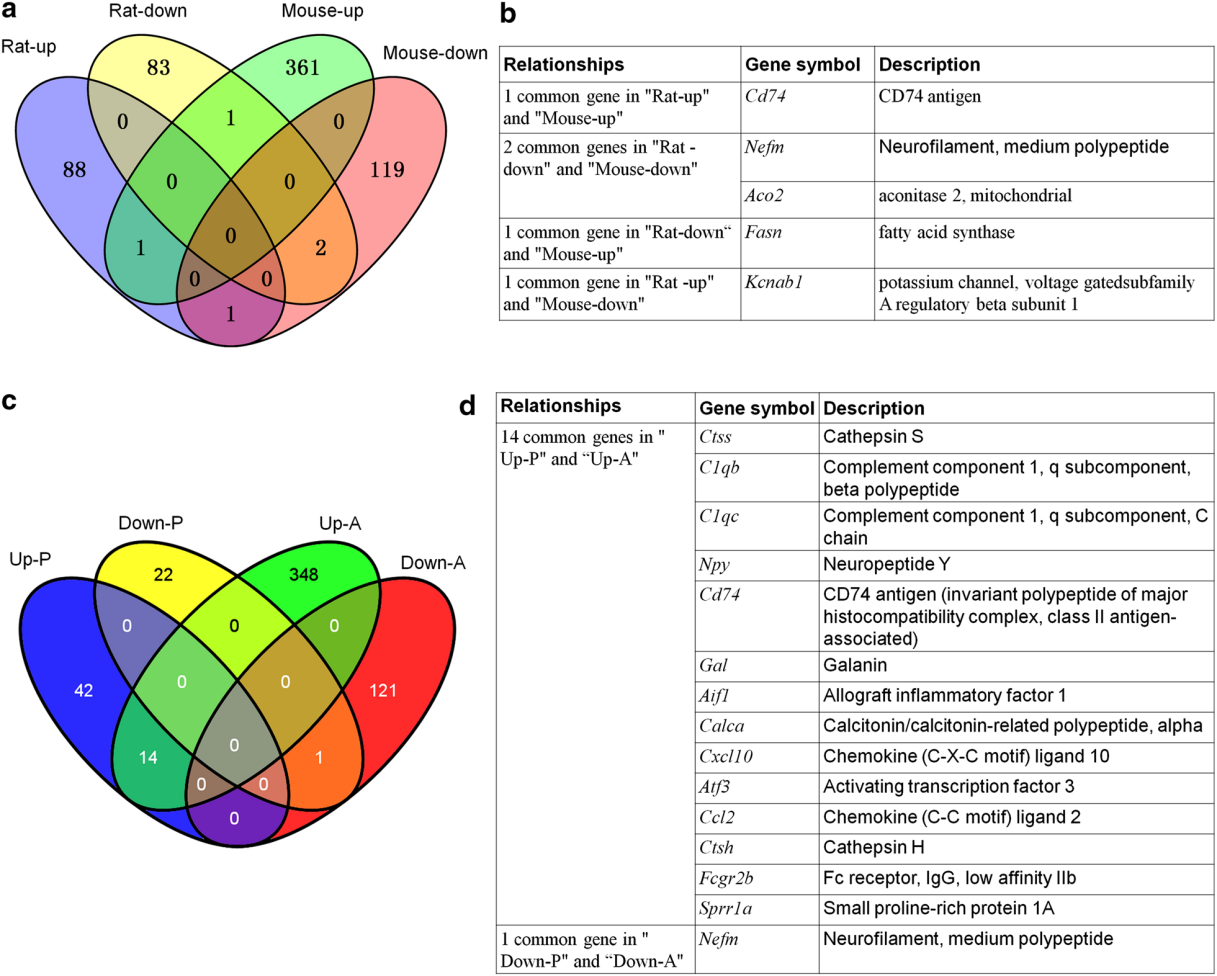
Gene overlap analysis between the present data and previously published microarrays in pain model. **a** Venn diagram showing the number of common up- and down-regulated genes in our present mice model (mice-up, mice-down) and previously published rat model (rat-up, rat-down) after SNL. Only three genes were shared with the same tendency between the two microarray experiments. **b** The detailed information of the overlap genes that were significantly regulated in both the mice and rat spinal cord. **c** Venn diagram showing the overlap between gene-sets of our present data and previously published microarrays (*Up-P* up-regulated genes of the previous studies, *Down-P* down-regulated genes of the previous studies, *Up-A* up-regulated genes of the author’s data, *Down-A* down-regulated genes of the author’s data). **d** The detailed information of 14 up-regulated and 1 down-regulated overlapped genes between our present data and previously published microarrays.

Previous studies have shown differential gene expression profile in the spinal cord in rats with neuropathic pain [30, 31]. In order to compare neuropathic pain-associated gene expression patterns in mice and rats, we did the overlap analysis between other’s microarray data from rat [30] and our current data from mice (Figure 7a). LaCroix-Fralish et al. reported that 88 genes were upregulated and 83 genes were downregulated in the spinal cord 7 days after L5 nerve root ligation in rats [30]. Surprisingly, compared to 361 up-regulated genes and 119 down-regulated genes in mouse, only 1 gene (*Cd74*) was upregulated and 2 genes (*Nefm*, *Aco2*) were downregulated in both rats and mice (Figure 7b). In addition, we compared our array data with 79 significantly regulated genes which were identified by meta-analysis from 20 independent microarray experiments from rats and mice after tissue inflammation or nerve injury [2]. We observed an overlap of 15 genes with the meta-analysis dataset (Figure 7c). These genes included 14 up-regulated genes (*Ctss*, *C1qb*, *C1qc*, *Npy*, *Cd74*, *Gal*, *Aif1*, *Calca*, *Cxcl10*, *Atf3*, *Ccl2*, *Ctsh*, *Fcgr2b* and *Sprr1a*) and 1 down-regulated gene (*Nefm*) (Figure 7d).

### Relational analysis of lncRNAs and mRNAs

**Figure 8:**
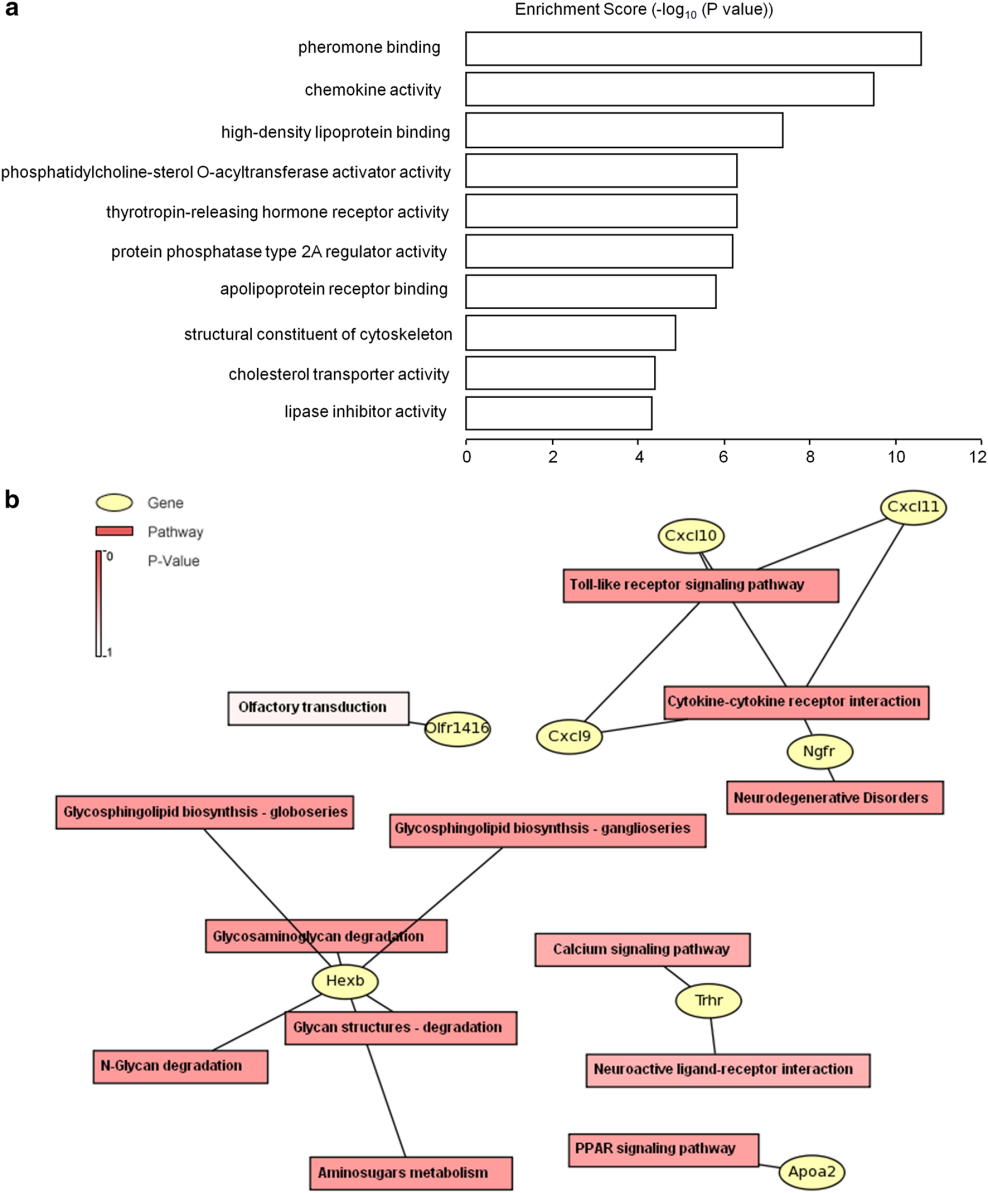
Function prediction of DE lncRNAs with related mRNAs. **a** Molecular function enrichment analysis of DE lncRNAs-related mRNAs. The enrichment scores (−log_10_ (P-value)) of the GO molecular function were shown in the *histogram*. **b** Gene-pathway network graph of DE lncRNAs-related mRNAs from Table 3. The DE lncRNAs-related genes and the corresponding pathways were shown in the *circles* and *boxes*, respectively. The *color* of pathway terms is defined by the enrichment P value.

As some lncRNAs have been suggested to play key roles in regulating the expression of their neighboring or overlapping genes in genome wide, we further screened out DE mRNAs related to DE lncRNAs based on their location distributions on mouse chromosomes by UCSC Genome Browser. In the spinal cord, there are 39 DE lncRNA-mRNA pairs for 35 DE lncRNAs and 35 DE mRNAs. Among them, 32 pairs exhibited coordinated expression changes, and 7 pairs were non-coordinated, which may suggest a complex and various regulatory mechanisms across different lncRNAs and their target mRNAs. Intriguingly, all the seven non-coordinated lncRNA-mRNA pairs belong to intergenic lncRNA-mRNA pairs (Table 3). Further GO and pathway analysis showed that the high enriched molecular functions include pheromone binding, chemokine activity, high-density lipoprotein binding, and phosphatidylcholine-sterol *O*-acyltransferase activator activity (Figure 8a). Based on gene-pathway network graph analysis, we found that the DE mRNAs from lncRNA-mRNA pairs, such as *Cxcl9* (chemokine (C-X-C motif) ligand 9), *Cxcl10* (chemokine (C-X-C motif) ligand 10), *Cxcl11* (chemokine (C-X-C motif) ligand 11), *Trhr* (thyrotropin releasing hormone receptor), and *Apoa2* (apolipoprotein A-II), might involve in toll-like receptor signaling pathway, calcium signaling pathway, and PPAR signaling pathway (Figure 8b; Table 3), which have been proven to be involved in neuropathic pain pathogenesis [32–34].

**Table 3:** DE lncRNAs and their neighboring or overlapping DE mRNAs

LncRNAs			Relationship	mRNAs			Function prediction of DE lncRNAs with related mRNAs	
Sequence name	Fold change	Regulation		GeneSymbol	Fold change	Regulation	Molecular Function	Pathway
*ENSMUST00000160110*	3.9130898	Down	Antisense overlap	*Phtf1*	2.1720073	Down	GO:0003677 DNA binding	
*AK136749*	2.089502	Up	Antisense overlap	*Asap2*	8.8652115	Up		
*ENSMUST00000121460*	11.624642	Up	Antisense overlap	*Mup2*	16.324926	Up	GO:0005215 transporter activity GO:0005550 pheromone binding	
*mouselincRNA1303+*	2.959626	Up	Intergenic	*Vmn1r54*	2.5373068	Up		
*MM9LINCRNAEXON12110+*	9.611986	Up	Intergenic	*Apoa2*	5.3063893	Up	GO:0005319 lipid transporter activity GO:0008035 high-density lipoprotein binding GO:0017127 cholesterol transporter activity GO:0042803 protein homodimerization activity GO:0046982 protein heterodimerization activity GO:0055102 lipase inhibitor activity GO:0060228 phosphatidylcholine-sterol *O*-acyltransferase activator activity	PPAR signaling pathway
*MM9LINCRNAEXON11813−*	2.2022471	Up	Intergenic	*Ngfr*	2.3073637	Up	GO:0005030 neurotrophin receptor activity GO:0048406 nerve growth factor binding	Neurodegenerative disorders Cytokine–cytokine receptor interaction
*C75950*	2.3177905	Up	Intergenic	*Gm5136*	2.2296717	Down		
*mouselincRNA1231−*	2.3548565	Up	Intergenic	*Hvcn1*	2.0545347	Up	GO:0005244 voltage-gated ion channel activity GO:0030171 voltage-gated proton channel activity	
*ENSMUST00000133243*	2.2177694	Up	Intergenic	*Uspl1*	2.747246	Up	GO:0004221 ubiquitin thiolesterase activity	
*MM9LINCRNAEXON11661+*	20.514269	Up	Intergenic	*Asap2*	8.8652115	Up		
*humanlincRNA1070+*	6.5686955	Up	Intergenic	*Vax2*	17.30259	Up	GO:0003700 transcription factor activity	
*humanlincRNA2255−*	6.4199057	Up	Intergenic	*Trhr*	2.1457152	Down		
*mouselincRNA1631+*	2.131738	Up	Intergenic	*Klhl15*	2.0060081	Up	GO:0005515 protein binding	
*humanlincRNA1443−*	4.366208	Up	Intergenic	*Igsf10*	7.495438	Up	GO:0005021 vascular endothelial growth factor receptor activity GO:0005515 protein binding GO:0005524 ATP binding	
*MM9LINCRNAEXON12110+*	9.611986	Up	Intergenic	*Dedd*	2.2202826	Down	GO:0003677 DNA binding GO:0005515 protein binding	
*MM9LINCRNAEXON10576−*	5.209898	Up	Intergenic	*Cxcl9*	5.6018896	Up	GO:0008009 chemokine activity	Cytokine–cytokine receptor interaction Toll-like receptor signaling pathway
*MM9LINCRNAEXON11308+*	3.7596319	Up	Intergenic	*Zfp654*	2.1100945	Down	GO:0003677 DNA binding GO:0008270 zinc ion binding	
*BM248967*	6.0079184	Up	Intergenic	*Dgkk*	3.3316648	Up	GO:0004143 diacylglycerol kinase activity	
*MM9LINCRNAEXON11616+*	2.5639145	Up	Intergenic	*Hexb*	2.0003252	Up	GO:0004553 hydrolase activity, hydrolyzing O-glycosyl compounds GO:0004563 beta-*N*-acetylhexosaminidase activity GO:0042803 protein homodimerization activity GO:0043169 cation binding GO:0046982 protein heterodimerization activity	*N*-Glycan degradation Aminosugars metabolism Glycosaminoglycan degradation Glycosphingolipid biosynthesis—globoseries Glycosphingolipid biosynthesis—ganglioseries Glycan structures—degradation
*uc008iab.1*	2.1543121	Down	Intergenic	*Fam160b1*	2.5731633	Up		
*MM9LINCRNAEXON12066−*	4.066157	Down	Intergenic	*Tnnt2*	3.0204759	Down	GO:0005200 structural constituent of cytoskeleton	
*MM9LINCRNAEXON10576−*	5.209898	Up	Intergenic	*Cxcl11*	2.7319772	Up	GO:0008009 chemokine activity	Cytokine–cytokine receptor interaction Toll-like receptor signaling pathway
*MM9LINCRNAEXON10576−*	5.209898	Up	Intergenic	*Cxcl10*	6.9877048	Up	GO:0008009 chemokine activity	Cytokine–cytokine receptor interaction Toll-like receptor signaling pathway
*AK054438*	2.3012707	Up	Intergenic	*Ifi202b*	9.431554	Up	GO:0005515 protein binding	
*MM9LINCRNAEXON10268−*	6.8239675	Up	Intergenic	*Irf8*	2.335659	Up	GO:0003700 transcription factor activity	
*MM9LINCRNAEXON11735+*	2.4868224	Down	Intergenic	*Ppp2r5c*	2.1656942	Down	GO:0008601 protein phosphatase type 2A regulator activity	
*DV650983*	2.0293975	Down	Intergenic	*Olfr1416*	2.2257524	Up	GO:0004984 olfactory receptor activity	Olfactory transduction
*MM9LINCRNAEXON11795+*	2.5727692	Down	Intergenic	*Cd68*	2.6843183	Up		
*MM9LINCRNAEXON11793+*	2.671865	Up	Intergenic	*Cd68*	2.6843183	Up		
*ENSMUST00000120184*	2.5531633	Down	Sense overlap	*Amy2b*	2.4439986	Down		
*uc007vpp.1*	2.1796808	Down	Sense overlap	*Trhr*	2.1457152	Down	GO:0004872 receptor activity GO:0004997 thyrotropin-releasing hormone receptor activity	Calcium signaling pathway Neuroactive ligand–receptor interaction
*uc009pmr.1*	3.20246	Down	Sense overlap	*Elmod1*	2.2530112	Down		
*uc007cua.1*	6.378551	Down	Sense overlap	*Tnnt2*	3.0204759	Down		
*ENSMUST00000040306*	4.188862	Down	Sense overlap	*H2-Ea-ps*	3.2939498	Down		
*uc008uzw.1*	2.2820547	Up	Sense overlap	*Laptm5*	2.1142242	Up		
*ENSMUST00000117412*	2.5116289	Up	Sense overlap	*Gm10147*	2.2881203	Up		
*ENSMUST00000119882*	3.1487308	Up	Sense overlap	*Gm10486*	2.4736855	Up		
*ENSMUST00000119882*	3.1487308	Up	Sense overlap	*Gm14819*	3.018787	Up		
*uc008tbm.1*	10.098583	Up	Sense overlap	*Mup17*	21.82689	Up

## Discussion

Chronic neuropathic pain is a somatosensory disorder caused by nerve injury or disease that affects the nervous system [35]. Evidence suggested that the particular patterns of gene expression at different levels of the nociceptive system play important roles in the development and maintenance of neuropathic pain [2, 36]. Over the past decades, the molecular mechanisms underlying neuropathic pain have been extensively studied; however, the pathophysiological process of pain is still vague. LncRNAs were recently shown to regulate gene expression [37] and traffic cellular protein complexes, genes, and chromosomes to appropriate locations [8]. Their function in regulating gene expression switching in the maintenance phase of neuropathic pain is poorly understood. In this study, we for the first time identified the global expression changes in lncRNAs and analyzed their characteristics and possible relation with coding genes in the spinal cord under neuropathic pain condition. The 24,833 lncRNAs were detected in the spinal cord of mice. Among them, 366 lncRNAs were up-regulated and 145 lncRNAs were down-regulated at 10 days after SNL. These DE lncRNAs are consistently altered in a high percentage of analyzed spinal cords from SNL and sham mice, suggesting that lncRNAs may be involved in neuropathic pain processing. So far, most DE lncRNAs have not been functionally characterized. Although it was still too early to translate this knowledge into the development of novel analgesic agents for better pain relief, these findings may likely provide novel insight into the molecular basis of pain.

In this study, the expression profiles of mouse genome-wide mRNAs were also detected using lncRNA Microarray Chip at the same time. Among DE mRNAs, the up-regulated mRNAs are far more numerous than the down-regulated in SNL samples, which reflects the emergence of new biology processes and pathways in pathological conditions. A number of reported pain-related genes, including *Cacna1g*, *Trpv1*, *Ccl5*, *Cx3cr1* and *Irf5* were dramatically increased after SNL. Moreover, a lot of other mRNAs, such as *Sprr1a*, *Anxa10*, *Kng1*, and *Gpr151* (G-protein-coupled receptor 151), whose functions are unclear in the spinal cord were also screened out. As the expression changes for some genes may be related to nerve damage and homeostatic responses to denervation, further studies are needed to identify whether they are involved in neuropathic pain processing.

Based on the GO term enrichment analyses of DE mRNA, we found that significantly enriched molecular functions and biological processes of up-regulated gene in SNL vs sham were mainly involved in chemokine activity, inflammation, and immunity. These findings are consistent with previous studies showing that neuroinflammation, manifested as infiltration of immune cells [38], activation of glial cells [39] and production of inflammatory mediators [40] in the peripheral and CNS, plays an important role in the induction and maintenance of chronic pain [41]. Additionally, our immunostaining of GFAP and IBA-1 showed dramatic glial activation in the spinal cord at 10 days after SNL. From significant pathway analyses of DE gene, the third most significant enriched pathway of the up-regulated genes in SNL vs sham is the toll-like receptor signaling pathway. Indeed, *Tlr2* [42], *Tlr4* [43], and *Tlr7* [44] have been implicated as potential therapeutic targets in neuropathic and other pain models. The data collectively indicate that anti-neuroinflammation may be an effective strategy for the treatment of neuropathic pain.

Previous studies utilizing cDNA microarrays to analyze gene expression profiles primarily focus on pain models in rats, rarely in mice [2]. The overlap analysis showed little overlap between rat and mice spinal cord gene expression patterns under neuropathic pain states, suggesting the species difference in gene expression. However, we found that there were 15 overlap genes between our current data and meta-analysis results reported by LaCroix-Fralish et al. [2]. These overlap genes including *Atf3*, *Sprr1al* and *Nefm* can be induced by nerve damage, which contribute to chronic pain [45–47]. In addition, gene ontology-based functional annotation clustering analyses of the previous gene chip study revealed strong evidence for regulation of immune-related genes in pain states, which was consistent with our data.

Although lncRNAs play important roles in the regulation of gene expression [48], there is a large gap between the number of existing lncRNAs and their known association with a particular molecular or cellular function [49]. Regulatory mechanisms and major functional principles of lncRNAs are complex and quite obscure. Unlike microRNA, there are no common languages that can be used to predict lncRNAs’ target genes and function by their sequence information or secondary structure. Accumulating evidence suggests that a number of lncRNAs function locally to activate or repress their neighboring or overlapping genes’ expression [18, 27, 50]. In this study, we found that intergenic lncRNAs (lincRNAs) were the largest category in all DE lncRNAs after SNL. In reality, lincRNAs are found to be conserved across multiple vertebrate species [51] and perform important functions in many cellular processes, from cell proliferation to cancer progression [52]. Furthermore, lincRNAs can function through different types of mechanisms, including *cis* or *trans* transcriptional regulation, translational control, splicing regulation, and other post-transcriptional regulation [33]. We examined whether their neighboring or overlapping protein-coding genes in the genome are simultaneously DE in the spinal cord after SNL, and found that there are 39 DE lncRNA-mRNA pairs. Our further analysis showed that an up-regulated lincRNA, *MM9LINCRNAEXON10576*− in the spinal cord after SNL was found to be located near *Cxcl10*, *Cxcl9* and *Cxcl11* gene cluster in mice chromosome 5. All the four RNAs have the same expression trends and increased more than twofold after SNL. Recently, studies using animal models have shown that upregulation of chemokines in the spinal cord play a vital role in the development and maintenance of chronic pain [41, 53, 54]. Indeed, recent research found that *Cxcl10* and its receptor *Cxcr3* were involved in inflammatory pain and cancer pain [55–57]. Therefore, lncRNA *MM9LINCRNAEXON10576*− may contribute to neuropathic pain through regulation of chemokines *Cxcl10*, *Cxcl9* and *Cxcl11*.

In our microarray results, 12 DE mRNA have their corresponding DE sense-overlap lncRNAs, and the change patterns of these lncRNA were same as that of their accompanying protein-coding genes. Di et al. found that a sense-overlap lncRNA arising from the CCAAT/enhancer-binding protein alpha (*Cebpa*) gene locus can bind to DNA methyltransferase 1 (DNMT1) and prevent *Cebpa* gene locus methylation, then to increase the expression of *Cebpa* gene. Their deep sequencing of transcripts associated with DNMT1 combined with genome-scale methylation and expression profiling extend the generality of this finding to numerous gene loci. [27]. Given that the 12 DE mRNA and their DE sense-overlap lncRNAs were both increased after SNL, it’s possible that the DE sense-overlap lncRNAs regulate the expression of their sense-overlapping mRNAs via demethylation after SNL.

## Conclusion

Our results demonstrated that lncRNA transcripts were highly enriched and hundreds of lncRNAs were differentially expressed in the spinal cord after SNL. Dozens of DE lncRNAs were observed to have neighboring or overlapping DE mRNAs in genome. These lncRNAs may locally regulate their related protein-genes expression and play key roles in the pathogenesis of neuropathic pain. Further studies are required to clarify the molecular and cellular functions of DE lncRNAs and determine whether they can serve as novel analgesic targets in neuropathic pain.

## Methods

### Animals and surgery

Adult male ICR mice (male, 8 weeks) were maintained on a 12:12 light–dark cycle at a room temperature of 22 ± 1°C with free access to food and water. The experimental procedures were approved by the Animal Care and Use Committee of Nantong University and performed in accordance with the guidelines of the International Association for the Study of Pain. To produce a SNL, animals were anesthetized with isoflurane and the L6 transverse process was removed to expose the L4 and L5 spinal nerves. The L5 spinal nerve was then isolated and tightly ligated with 6-0 silk threads [58]. For sham operations, the L5 spinal nerve was exposed but not ligated.

### Behavioral test

Animals were habituated to the testing environment daily for at least 2 days before baseline testing. The room temperature remained stable for all experiments. For testing mechanical sensitivity, animals were put in boxes on an elevated metal mesh floor and allowed 30 min for habituation before examination. The plantar surface of each hindpaw was stimulated with a series of von Frey hairs with logarithmically incrementing stiffness (0.02–2.56 g, Stoelting, Wood Dale, IL, USA), presented perpendicular to the plantar surface (2–3 s for each hair). The 50% paw withdrawal threshold was determined using Dixon’s up-down method [59]. For testing heat sensitivity, animals were put in plastic boxes and allowed 30 min for habituation. Heat sensitivity was tested by radiant heat using Hargreaves apparatus (IITC Life Science Inc., Woodland Hills, CA, USA) and expressed as paw withdrawal latency (PWL). The radiant heat intensity was adjusted so that basal PWL is between 10 and 14 s, with a cutoff of 18 s to prevent tissue damage.

### Immunohistochemistry

At 10 days after SNL or sham-operation, animals were deeply anesthetized with isoflurane and perfused through the ascending aorta with PBS followed by 4% paraformaldehyde with 1.5% picric acid in 0.16 M PB. After the perfusion, the L4–L5 spinal cord segments were removed and postfixed in the same fixative overnight. Spinal cord sections (30 μm, free-floating) were cut in a cryostat. The sections were first blocked with 5% goat serum for 2 h at room temperature. The sections were then incubated overnight at 4°C with the following primary antibodies: GFAP antibody (mouse, 1:6,000; Millipore, Billerica, MA, USA), IBA-1 antibody (Mouse, 1:3,000, Serotec, Kidlington, UK). The sections were then incubated for 2 h at room temperature with FITC-conjugated secondary antibodies (1:1,000, Jackson ImmunoResearch). The stained sections were examined with a Leica fluorescence microscope, and images were captured with a CCD Spot camera.

### Tissue collection and RNA isolation

We prepared nine mice for SNL and nine mice for sham-operation. At 10 days after operation, the animals were deeply anesthetized with isoflurane and perfused through the ascending aorta with saline. After the perfusion, the L4–L5 spinal cord segments were collected. Total RNA was extracted from the spinal cord dorsal horn tissue using Trizol reagent (Invitrogen, Carlsbad) according to the manufacturer’s protocol. The RNA concentration and purity were assayed by the absorbance values at 260 and 280 nm using the NanoDrop 1000 Spectrophotometer (Thermo). RNA integrity was checked by electrophoresis on 2% (m/v) agarose gels. After these testing, equal mRNA from three mice under the same treatment was mixed as one sample. Therefore, six samples (3 for SNL and 3 for sham) were sent for microarray analysis.

### Microarray assay

The gene chip of the mouse lncRNA microarray V2.0 (8 × 60K, Arraystar), which includes 25,376 lncRNA probes and 31,423 coding gene probes, was used in the experiments. The total RNAs of sham and SNL groups were individually hybridized with gene chips. Briefly, RNA was purified from 1 μg total RNA after removing rRNA. The RNA sample was then transcribed into fluorescent cRNA along the entire length of the transcripts without 3′ bias utilizing random primers. The labeled cRNAs were hybridized to mouse lncRNA microarray. Finally, arrays were scanned by Agilent Scanner G2505B. The array images were analyzed by Agilent Feature Extraction software (version 10.7.3.1). The GeneSpring GX v11.5.1 software package (Agilent Technologies) was utilized to analyze quintile normalization and subsequent data processing. The microarray hybridization was carried out by Kangchen Bio-tech, Shanghai, China.

### Bioinformatics analysis

Differentially expressed lncRNAs and mRNAs with statistical significance were identified through Volcano Plot filtering. The threshold used to screen up- or down-regulated RNAs was fold-change >2.0 (P < 0.05). Hierarchical clustering was carried out by Cluster 3.0, and the heat maps were generated in Java Treeview. The DE mRNAs which were adjacent to or overlap with the DE lncRNAs were recognized as DE lncRNAs related mRNAs using UCSC Genome Browser. The differentially expressed mRNAs or DE lncRNAs related mRNAs were analyzed by pathway annotation and gene ontology (GO) functional enrichment using CapitalBio^®^ Molecule Annotation System V3.0 (MAS3.0). The −log_10_ (P-value) of the GO and pathway results were shown in the histogram.

### Real-time reverse transcription-polymerase chain reaction (RT-PCR)

The microarray results were confirmed by RT-PCR. Total RNA was extracted from the spinal cord tissue as described above and total RNA was reverse transcribed using random hexamers primer (TaKaRa Bio Inc) according to the manufacturer’s description. The expression level of six genes was checked, including *Speer7*-*ps1*, *uc007pbc.1*, *ENSMUST00000171761*, *ENSMUST00000097503*, *Cyp2d9*, and *Mnx1*. The *Gapdh* was used as house-keeping gene. The sequences of all primers were shown in Table 4. RT-PCR was performed using the Fast Start Universal SYBR Green Master (TaKaRa Bio Inc) with 20-μl reaction system, according to the manufacturer’s protocol, in a Rotor-Gene 6000 instrument (Hamburg, Germany). The melting-curve analysis was performed in order to monitor the specificity of production. All experiments were replicated three times. The gene expression levels in the sham and SNL groups were analyzed with the 2^−∆∆CT^ method.

**Table 4:** Primer sequences used in Real-Time PCR

Sequence name	Primer sequence	Amplicon size (bp)
*Speer7-ps1*	F: 5′-CATGCTCTCATGCTCACCGA-3′	70
	R: 5′-TACGCTGTAGGACCAGAACAC-3′	
*uc007pbc.1*	F: 5′-CATCTAGACCCGTAACGCCC-3′	340
	R: 5′-TGGTAGGCAAGCATCCACAG-3′	
*ENSMUST00000171761*	F: 5’-TCGGAGACTTCTCTTCCGGT -3’	108
	R: 5′-AAGACAATGCAGATGGGGCA-3′	
*ENSMUST00000097503*	F: 5′-AGGTCATCCCACTTTGGTACAC-3′	77
	R: 5′-GAGTTTGGTTTGCGGGGTCT-3′	
*Cyp2d9*	F: 5′-TGTCTACCCTGCGCAACTTT-3′	71
	F: 5′-GTGATTGGCCTCCTTGGTCA-3′	
*Mnx1*	F: 5′-GAACACCAGTTCAAGCTCAACA-3′	129
	R: 5′-GCTGCGTTTCCATTTCATTCG-3′	
*Gapdh*	F:5′-TGTTCCTACCCCCAATGTG-3′	129
	R:5′-GTGTAGCCCAAGATGCCCT-3′	

### Statistical analysis

The behavioral data were analyzed by two-way analysis of variance. The RT-PCR results were reported as mean ± SEM and analyzed by the one-way analysis of variance followed by Tukey’s multiple comparison test. The criterion for statistical significance was P < 0.05.

## Abbreviations

*Anxa10*: annexin A10
*Apoa2*: apolipoprotein A-II
*Atf3*: activating transcription factor 3
*Cacna1g*: calcium channel, voltage-dependent, T type, alpha 1G subunit
*Ccl5*: chemokine C-C motif ligand 5
*Cebpa*: CCAAT/enhancer-binding protein alpha
CNS: central nervous system
*Cyp2d9*: cytochrome P450, family 2, subfamily d, polypeptide 9
*Cx3cr1*: chemokine (C-X3-C) receptor 1
DE: differentially expressed
*Dnmt1*: DNA methyltransferase 1
GO: gene ontology
*Gpr151*: G-protein-coupled receptor 151
*Irf5*: interferon regulatory factor 5
*Kng1*: kininogen 1
miRNA: microRNA
lncRNA: long non-coding RNA
*Mnx1*: motor neuron and pancreas homeobox 1
*Nefm*: neurofilament, medium polypeptide
PPAR: peroxisome proliferator-activated receptor
SNL: spinal nerve ligation
*Sprr1a*: small proline-rich protein 1A
*Tagap*: T cell activation Rho GTPase-activating protein
*Trhr*: thyrotropin releasing hormone receptor
*Trpv1*: transient receptor potential cation channel, subfamily V, member 1
*Zfp236*: zinc finger protein 236
